# Potential harms associated with routine collection of patient sociodemographic information: A rapid review

**DOI:** 10.1111/hex.12837

**Published:** 2018-10-19

**Authors:** Jennifer Petkovic, Stephanie L. Duench, Vivian Welch, Tamara Rader, Alison Jennings, Alan J. Forster, Peter Tugwell

**Affiliations:** ^1^ Bruyère Research Institute University of Ottawa Ottawa Ontario Canada; ^2^ Canadian Agency for Drugs and Technologies in Health Ottawa Ontario Canada; ^3^ Clinical Epidemiology Program Ottawa Hospital Research Institute Ottawa Ontario Canada; ^4^ Department of Medicine University of Ottawa Ottawa Ontario Canada; ^5^ School of Epidemiology and Public Health University of Ottawa Ottawa Ontario Canada

## Abstract

**Background:**

Health systems are recommended to capture routine patient sociodemographic data as a key step in providing equitable person‐centred care. However, collection of this information has the potential to cause harm, especially for vulnerable or potentially disadvantaged patients.

**Objective:**

To identify harms perceived or experienced by patients, their families, or health‐care providers from collection of sociodemographic information during routine health‐care visits and to identify best practices for when, by whom and how to collect this information.

**Search Strategy:**

We searched OVID MEDLINE, PubMed “related articles” via NLM and healthevidence.org to the end of January 2018 and assessed reference lists and related citations of included studies.

**Inclusion Criteria:**

We included studies reporting on harms of collecting patient sociodemographic information in health‐care settings.

**Data Extraction and Synthesis:**

Data on study characteristics and types of harms were extracted and summarized narratively.

**Main Results:**

Eighteen studies were included; 13 provided patient perceptions or experiences with the collection of these data and seven studies reported on provider perceptions. Five reported on patient recommendations for collecting sociodemographic information. Patients and providers reported similar potential harms which were grouped into the following themes: altered behaviour which may affect care‐seeking, data misuse or privacy concerns, discomfort, discrimination, offence or negative reactions, and quality of care. Patients suggested that sociodemographic information be collected face to face by a physician.

**Discussion and Conclusions:**

Overall, patients support the collection of sociodemographic information. However, harms are possible, especially for some population subgroups. Harms may be mitigated by providing a rationale for the collection of this information.

## 1 BACKGROUND

Hospitals and health clinics routinely collect information from their patients for administrative reasons and medical records. This information provides basic information about patients and health concerns. However, if additional sociodemographic data are obtained, it could also be used to inform strategies and policies to improve health equity, defined as the absence of differences in health outcomes that are reasonably avoidable.^1^, ^2^

Population characteristics that may contribute to health inequities can be captured using the acronym PROGRESS‐Plus, which stands for place of residence, race/ethnicity/culture/language, occupation, gender/sex, religion, education, socio‐economic status, social capital and “plus” to capture additional characteristics, such as context‐relevant personal characteristics (eg age), features of relationships and time‐dependent characteristics.^3^, ^4^, ^5^ Routine capture of this information will enable the development of specific solutions to address service gaps to these potentially disadvantaged populations. Health systems need to consider the optimal method for routinely capturing this information. For hospitals, routine data capture works best if it can be incorporated into existing work flows.^6^, ^7^

For some PROGRESS‐Plus characteristics, there are strong clinical indications to obtain this information to guide clinical decisions. For these concepts, it is sensible to collect the information during routine clinical processes, such as during patient registration or during the provision of a medical history by the patient to a provider. However, for several PROGRESS characteristics, specifically race/ethnicity/culture/language, religion and income, the relevance of the information for clinical decision making may not be apparent to patients. Self‐reported sociodemographic information is more reliable than health‐care provider observation‐determined which may lead to stereotyping based, for example, on name and skin colour.^8^ Therefore, collection of information would be most useful when collected directly from patients, but studies have found that the collection of this information may cause patient distress, especially for patients from potentially disadvantaged or vulnerable populations.^6^, ^7^ In addition, because of the uncertainty of the immediate clinical benefits derived from the collection of this type of information, it may be difficult to obtain and the collection itself could interfere with the trust relationship between patients and their providers.

We conducted a rapid review to identify the potential harms associated with the collection of race/ethnicity/culture/language, religion and income information as well as best practices for how, when and by whom these data should be collected.

## 2 OBJECTIVE

The objectives of this rapid review are to:

- Identify potential or actual harms experienced by patients or their families when they are asked to provide information about their race/ethnicity/culture/language, religion and income during routine health‐care visits.
- Identify clinician's concerns with the potential harms experienced by patients or their families when they are asked to provide information about their race/ethnicity/culture/language, religion and income during routine health‐care visits.
- Identify best practices for the routine collection of sociodemographic data to reduce the risks of potential harms for patients and their families.

## 3 METHODS

We conducted a “rapid review” using accelerated systematic review methods based on the Cochrane Handbook for Systematic Reviews of Interventions, and the methods recommended by the Cochrane Rapid Reviews Methods Group and the Knowledge Synthesis Group at the Ottawa Hospital Research Institute.^9^, ^10^, ^11^ A protocol for this work was developed a priori and published on the Campbell and Cochrane Equity Website (https://methods.cochrane.org/equity/projects/sociodemographic-data-collection).

### 3.1 Search strategy

We developed and tested a search strategy (Appendix S1) and searched the following databases on 30‐31 January 2018:

- MEDLINE via OVID (1946 to 31 January 2018),
- PUBMED via NLM “related articles” search in PUBMED using five “gold standard” articles as seed papers and
- Healthevidence.org.

No methodological filters, date limitations or language restrictions were applied. We also screened the reference lists of included studies and their related citations using Scopus to retrieve the abstracts.

### 3.2 Study selection

The titles and abstracts of de‐duplicated citations were screened using an accelerated method in which a study assessed as relevant was included for full‐text screening without being reviewed by a second assessor, but studies assessed as not relevant were assessed by another member of the author team. The full‐text papers identified as potentially relevant were screened independently, in duplicate, by two members of the author team.

Eligibility was assessed using the following criteria.

*Population*: Patients presenting to health‐care providers including hospitals or clinics. It is important to note that some studies surveyed the general populations’ perceptions on the collection of sociodemographic information in health‐care settings if they were to present to a health‐care provider. For the purposes of this report, these participants will be referred as patients. We excluded health‐care plan settings (eg data collected by health insurance providers).
*Interventions*: Routinely solicited information on race/ethnicity/culture/language, religion and/or income—by survey or by direct questioning.
*Comparison*: No comparator was required for this review.
*Outcomes*: Provider's, patient's and/or family's perception or experience of adverse outcomes including a perception of persecution; a perception the information will be used for ulterior purposes; and other reasons for failure to provide information.

### 3.3 Data collection and synthesis

Data extraction forms were developed and tested using Excel. Data were extracted for country, study design, data collection method, type of data collected, setting, population and outcome. Data were extracted by one reviewer and verified by a second reviewer. Evidence was synthesized in data summary tables.

## 4 RESULTS

### 4.1 Results of search

**Figure 1:**
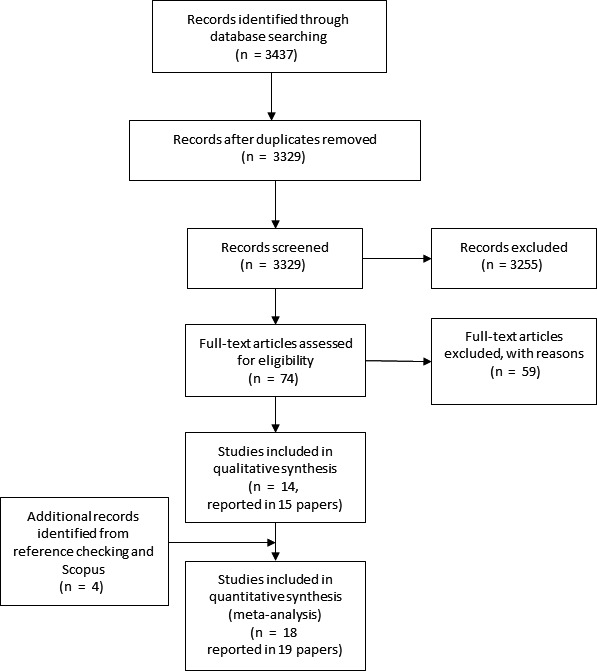
PRISMA flow diagram

The search identified 3437 records (Figure 1). After duplicates were removed, 3329 titles and abstracts were assessed for eligibility. We assessed the full text of 74 studies from which 59 were excluded because they did not obtain primary data, they did not discuss possible negative outcomes for patients from the collection of race/ethnicity/culture/language, religion or income information, or the data were collected outside of the health‐care setting (eg for health insurance plans). We identified four additional studies through searching the reference lists of included studies and using the related citation function in Scopus. A total of 18 studies reported in 19 papers were included in this review.

### 4.2 Study characteristics

Of the 18 studies included in this review, 11 (61.1%) exclusively assessed patients’ perceptions or experiences of the harms associated with the routine collection of sociodemographic data.^12^, ^13^, ^14^, ^15^, ^16^, ^17^, ^18^, ^19^, ^20^, ^21^, ^22^ Four (22.2%) studies exclusively assessed providers’ perceptions of the potential harms for patients from the routine collection of sociodemographic data.^23^, ^24^, ^25^, ^26^ The remaining three (16.7%) studies assessed both patients’ perceptions or experiences of harms and providers’ perceptions of harms.^27^, ^28^, ^29^

The included studies were conducted in the USA (66.7%),^12^, ^13^, ^15^, ^17^, ^21^, ^22^, ^23^, ^25^, ^26^, ^27^, ^28^, ^29^ Canada (27.8%)^16^, ^18^, ^19^, ^20^, ^21^, ^30^ and the UK (11.1%).^14^, ^24^ Three studies employed a mixed‐methods design,^16^, ^21^, ^27^ eight studies used a quantitative design,^12^, ^13^, ^18^, ^19^, ^20^, ^24^, ^26^ and the remaining seven used a qualitative design.^14^, ^17^, ^22^, ^23^, ^28^, ^29^, ^30^ Three studies included a large variety of racial/ethnic groups including white, black/African American, Latino/Hispanic, Asian and multiracial participants.^12^, ^13^, ^15^ One study included only South Asians.^14^ Other studies included the general population^18^, ^20^ and/or health‐care service users.^16^, ^19^ One study (reported in two papers) included a range of participants including community leaders, health‐care workers, health‐care service users and health policy decision makers.^21^, ^30^ Health‐care representatives (eg hospital executives and physicians) were participants in the remaining studies.^17^, ^22^, ^23^, ^24^, ^25^, ^26^, ^27^, ^28^, ^29^ The characteristics of the included studies are described in Table 1, and a map of the potential harms reported in each study is provided in Table 2.

**Table 1:** Characteristics of included studies

Reference	Country and Region	Population	Type of data collected	Data collection method
Baker et al. (2005)^12^	USA: Illinois	English‐speaking general internal medicine patients (n = 220) Mean age of participants was 44 y. 66.7% were female. 41.4% white, 34.1% black/African American, 9.1% Latino/Hispanic, 4.6% Asian, 8.2% multiracial/ethnic and 2.7% other or refused Response rate was 59.0%. Refusal rates were highest for whites and lowest for Hispanics	Patients’ perceptions on the collection of race/ethnicity information from clerks in hospitals and clinics	In‐person survey
Baker et al. (2007)^13^	USA: California	Californians (n = 563). Mean age of participants was 47.1 y. 62.7% were female. 18.7% white, 17.2% black, 35.4% Hispanic/Latino, 22.9% Asian, 5.2% multiracial and 0.7% other. Response rate was 39.6%	Californians’ perceptions for the collection of race/ethnicity and language information from clerks in hospitals and clinics.	Telephone survey
Hasnain‐Wynia et al. (2004)^27^	USA: Nationwide	Site visits: Consortium hospitals (n = 6) to talk to key clinical, research, operation information technology, admitting, patient registration and quality assurance staff. Survey: Hospitals nationwide (n = 250). The response rate was 27%	Hospitals’ current practices on and experiences with race/ethnicity and language data collection	Site visits and paper survey
Hasnain‐Wynia et al. (2010)^23^	USA: Nationwide	Health‐care practices in the USA with 5 or fewer physicians (n = 20)	Physicians’ perceptions on the collection of race/ethnicity and primary language information in health‐care practices	Telephone semi‐structured interviews
Iqbal et al. (2012)^14^	UK	South Asians originating from Pakistan, India and Bangladesh (n = 36)	South Asians’ perceptions and experiences on the collection of ethnicity, language, religion and culture information in a health‐care setting.	Focus groups
Iqbal et al. (2012)^24^	UK: England and Wales	Clinicians (n = 7), managers (n = 5), nurses (n = 5), information scientists (n = 6) and other staff involved in collecting or using ethnicity data in a health‐care setting (n = 7)	Health‐care staff's perceptions and experiences of ethnicity data collection in health‐care settings	Online survey
Jorgensen et al. (2010)^28^	USA: Massachusetts	Hospitals (n = 28) to talk to senior executives from the following areas: patient access and registration (n = 8); community, diversity and disparities (n = 7); quality, safety and performance (n = 6); information technology systems (n = 4); and finance (n = 3) Response rate for hospitals was 50%	Hospital senior executives reported patient perceptions and experiences with the collection of race/ethnicity and language information in hospitals	Semi‐structured telephone interviews
King et al. (2008)^25^	USA: Nationwide	Experts in racial/ethnic disparities in health care, quality improvement, implementation research and organization excellence (n = 20)	Experts’ perspectives on reducing racial/ethnic disparities	Forum
Kandula et al. (2009)^15^	USA: California	Californians (n = 480) Mean age of participants was 47 y. 61.7% were female. 21.0% white, 17.1% black, 36.0% Hispanic/Latino, 20.4% Asian and 5.4% multiracial Response rate was 39.6%	Californians’ perceptions on the collection of race/ethnicity information from clerks in hospitals and clinics	Telephone survey
Kirst et al. (2013)^16^	Canada: Ontario	Public opinion survey: Ontarians 18 y of age and older (n = 1306). 85% were over the age of 35, and 15% identified as an ethnic or cultural minority. Response rate was 8.2%. In‐depth interviews: Individuals who had used health‐care services within the last 12 mo and lived in Toronto (n = 34). 56% of participants were female, 85% were over the age of 35, and 26% identified as an ethnic or cultural minority	Ontarians’ and Toronto service users’ perceptions on the collection ethnicity, preferred language and household income information in health‐care settings.	Telephone survey and in‐depth in‐person interviews.
Lee et al. (2016)^17^	USA	N/A	Experiences and challenges with collecting race, ethnicity and language information.	Case study
Lofters et al. (2011)^18^	Canada: National	Canadians aged 18 y or older (n = 1005). 51.3% of participants were females, and 13.5% were ethnic/cultural minorities. Response rate was 3.1%.	Canadians’ perceptions on the importance of and concerns with the collection of ethnicity, preferred language and household income information in hospitals.	Telephone survey
Nerenz et al. (2004)^26^	USA: Nationwide	Hospitals nationwide (n = 262) The response rate was 26.2%	American hospitals’ current practices on the collection of race/ethnicity information	Paper survey
Pinto et al. (2016)^19^	Canada: Ontario	Patients 16 y of age or older from the family practice unit at St. Michaels hospital (n = 407) The response rate for each question ranged from 84% to 100%. Prefer not to answer responses occurred the most for the collection of income (10.1%)	Patients’ experiences completing a hospital sociodemographic survey with questions related to language, race, religion and income	iPad survey at family practice
Quan et al. (2006)^20^	Canada: Calgary	Individuals from Calgary aged 18 y or older (n = 2799) Response rate was 55%	Perceptions on the collection of ethnicity in hospitals	Telephone survey interview
Thorlby et al. (2011)^29^	USA: Nationwide	Senior managers, senior clinicians and data analysts from hospitals (n = 3), health plans (n = 3) and community health centres (n = 2) Response rate of organizations was 72.7%	Health‐care organizations’ current practices on the collection of race/ethnicity information	Case study and semi‐structured in‐person interviews
Varcoe et al. (2009)^21^	Canada: Western Canada	Focus groups: Diverse set of community leaders serving on advisory committees for the health authority (n = 18) Interviews: Health‐care workers who administered/were considering administering an ethnic identity question (n = 16) Semi‐structured interviews: Patients seeking health services in a subacute area of a large urban emergency department or a community health centre (n = 60) In‐depth interviews: Health policy decision makers from Western Canada who were responsible for addressing health equity issues (n = 10)	Community leaders, health‐care workers, patients and health policy decision makers’ perceptions and experiences with the collection of race/ethnicity information in a health‐care setting	Focus groups and semi‐structured and in‐depth interviews
Wilson et al. (2013)^22^	USA	N/A	Implementation, lessons learned and experiences from collecting race, ethnicity and language	Case study

**Table 2:** Overview of the patient and provider perceived or experienced harms by citation

	Perception or experience	Altered behaviour	Discomfort	Discrimination	Misuse/privacy concerns	Offence/other negative reactions	Quality of care
Baker et al. (2005)^12^	Perception	Patients	Patients	Patients			
Baker et al. (2007)^13^	Perception		Patients	Patients	Patients		
Hasnain‐Wynia et al. (2004)^27^	Experience		Patients	Providers	Providers	Patients Providers	Providers
Hasnain‐Wynia et al. (2010)^23^	Perception		Providers		Providers		
Iqbal et al. (2012)^14^	Perception and experience		Patients	Patients		Patients	
Iqbal et al. (2012)^24^	Perception					Providers	
Jorgensen et al. (2010)^28^	Perception and experience					Patients Providers	Patients Providers
Kandula et al. (2009)^15^	Perception		Patients	Patients	Patients		
King et al. (2008)^25^	Perception					Providers	
Kirst et al. (2013)^16^	Perception		Patients	Patients	Patients		Patients
Lee et al. (2016)^17^	Experience		Patients				
Lofters et al. (2011)^18^	Perception		Patients				Patients
Nerenz et al. (2004)^26^	Perception			Providers	Providers	Providers	Providers
Pinto et al. (2016)^19^	Experience		Patients				
Quan et al. (2006)^20^	Perception		Patients				
Thorlby et al. (2011)^29^	Perception					Providers	
Varcoe et al. (2009)^21^	Perception and experience	Patients		Patients	Patients	Patients	Patients
Wilson et al. (2013)^22^	Experience					Patients	

Results are presented in three categories: (a) patient perceptions or experiences, (b) provider perceptions and (c) patient recommendations for sociodemographic data collection.

### 4.3 Patients’ perceptions or experiences of harms

All 13 studies reporting patients’ perceptions or experiences assessed the harms associated with the collection of race/ethnicity/culture/language data.^12^, ^13^, ^14^, ^15^, ^16^, ^17^, ^18^, ^19^, ^20^, ^21^, ^22^, ^27^, ^28^, ^30^ Three studies assessed the harms associated with the collection of income data,^16^, ^18^, ^19^ and two studies assessed the harms associated with the collection of religion data.^14^, ^19^ Six studies reported patient perceptions,^12^, ^13^, ^15^, ^16^, ^18^, ^20^, ^30^ four reported patient experiences,^17^, ^18^, ^22^, ^27^ and three reported both patient perceptions and experiences.^14^, ^21^, ^28^

An overview of the various harms perceived or experienced by patients in each citation is presented in Table 2. A data summary of patient perceived or experienced harms is found in Table 3. We have grouped the potential perceived or experience harms into the following themes: altered behaviour, discomfort, discrimination, misuse and privacy concerns, offence and negative reactions, and quality of care.

**Table 3:** Patients’ perceptions or experiences of harms

Reference	Patient outcomes
Baker et al. (2005)^12^	79.9% of participants somewhat or strongly agreed that hospitals and clinics should collect information on race and ethnicity. Reported harms include the following:
	Altered Behaviour
	14.1% of participants would be less likely go to a hospital or clinic that records race/ethnicity information, especially Hispanics (26.3%) and blacks (18.5%).
	Discomfort
	21.8% of participants were moderately comfortable, and 15.5% were uncomfortable providing race/ethnicity information to a clerk. Black (24.3%) participants were more uncomfortable compared to whites (8.8%). Participants not fully comfortable providing race/ethnicity information to a clerk felt more comfortable providing this information to doctor or nurse.
	Discrimination
	19.8% of participants were somewhat concerned, and 31.4% were very concerned that the information collected could be used to discriminate against patients. Black (74.3%) participants were somewhat or very concerned more than whites (40.9%).
Baker et al. (2007)^13^	63.2% of participants somewhat or strongly agreed that HCPs should collect race/ethnicity information. 85.3% of participants somewhat or strongly agreed that HCPs should collect language information. Reported harms include the following:
	Discomfort
	21.8% of participants were moderately comfortable, and 17.2% were uncomfortable providing race/ethnicity information to a clerk. Hispanics (25.3%) and Chinese‐speaking Asians (17.5%) were more uncomfortable than whites (9.6%). Among participants whom English was not their preferred language, approximately half felt moderately comfortable or uncomfortable providing their English proficiency to a clerk. Hispanics (35.9%) were more likely to be uncomfortable than Chinese‐speaking Asians (13.8%).
	Discrimination
	46.3% of participants were somewhat or very worried that providing race/ethnicity and language information could be used to discriminate against them. Worry was higher among non‐white and multiracial/ethnic participants; 47.7% of Hispanics were very worried, and 23.8% were somewhat worried. There was also a worry that this information could be used to discriminate against others.
	Misuse and privacy concerns
	38.5% of participants were very worried, and 18.5% were somewhat worried that the government would use this information to find undocumented immigrants. The level of worry for misuse rose somewhat for whites, Hispanics, English‐speaking Asians and multiracial individuals.
Hasnain‐Wynia et al. (2004)^27^	Discomfort
	Hospital clerks indicated that patients felt uncomfortable providing race/ethnicity information.
	Offence/Negative reactions
	Patients felt offended by questions about race and ethnicity.
Iqbal et al. (2012)^14^	In general, participants thought that the collection of ethnicity data was important and were happy to disclose their religion and language as long as they did not perceive that they were being stereotyped. Reported harms include the following:
	Discomfort
	Many participants indicated concerns related to feeling discomfort if the purpose of collecting ethnicity data was not fully explained to them and feared being stereotyped.
	Discrimination
	11.1% of participants did not understand the need for ethnicity data collection as they did not believe that it was relevant to treatment or felt that it could be used to be discriminated against. When asked to report religion, some Muslims felt that they were being stereotyped with heightened awareness on terrorism.
	Offence/Negative reactions
	Many were dissatisfied about being asked on repeat visits and to report ethnicity without explanation. Participants reported negative experiences providing ethnicity data as they did not fit in any of the categories which resulted in them choosing “other” leading to feelings of frustration and insignificance.
Jorgensen et al. (2010)^28^	Although not frequently reported, executives from nine hospitals reported patient harms:
	Offence/Negative reactions
	Patients were very upset that they did not have the choice of Hispanic or Latino and were required to put white or black. Patients were angry, and some declined to answer.
	Quality of care
	Patients perceived that by providing their ethnicity, they would receive different care.
Kandula et al. (2009)^15^	61% of participants reported a high comfort level for giving registration staff information about their race/ethnicity. Reported harms include the following:
	Discomfort
	Hispanics and Asians were significantly less comfortable than whites providing their race/ethnicity information to a registration clerk. Comfort was significantly lower among those who experienced discrimination and perceived discrimination in general or in medical care.
	Discrimination
	Those who perceived discrimination in general and in medical care were more likely to worry that race/ethnicity information would be used to discriminate against them compared to those who did not perceive discrimination. Black, Hispanic and multiracial individuals were significantly more worried that race/ethnicity information could be used to discriminate against them compared to whites. Individuals with fair or poor self‐reported health were significantly less more worried than those with excellent/very good/good health. Increasing age and college graduate were associated with less worry for discrimination.
	Misuse and privacy concerns
	Only Hispanics were significantly more worried than whites that race/ethnicity information could be used by the government to find undocumented immigrants. Perceived discrimination in general and in medical care was associated with higher worry about the government using race/ethnicity information to find undocumented immigrants.
Kirst et al. (2013)^16^	49% of survey participants agreed that the collection of sociodemographic information in a health‐care setting was important. Reported harms from survey and interview participants include the following:
	Discomfort
	67% of participants felt uncomfortable disclosing household income. Participants expressed least comfort providing household income because they did not think that socio‐economic position should affect immediate health‐care delivery. 7% expressed discomfort to language collection. Older participants (55 y of age or older) were more likely to be uncomfortable providing ethnic and language background compared to younger participants (18‐34 y of age). Participants from ethnic or cultural minorities were more likely to be uncomfortable disclosing their preferred language than non‐minorities, and males were less comfortable than females. Participants were least comfortable disclosing information through existing government records and most comfortable disclosing information face to face with a physician.
	Discrimination
	Participants feared that income information could be used to judge, pity or discriminate against patients.
	Misuse and privacy concerns
	63% of survey participants were concerned about the misuse of data. Younger participants (18‐34 y of age) were more likely to be concerned than those 55 y of age or older, and females were more concerned than males. Over half of participants were concerned with security measures to prevent identity theft and privacy of personal information. Several interview participants were concerned with the confidentiality of collecting personal information, and for many, the security of this information was an indication on whether they were willing to disclose.
	Quality of care
	Participants believed that disclosing income information could negatively impact their care due to associated discrimination and judgement from the health‐care provider.
Lee et al. (2016)^17^	Discomfort
	Patients felt uncomfortable reporting their race and ethnicity.
Lofters et al. (2011)^18^	44% of participants agree with the importance of hospitals collecting sociodemographic data. Reported harms include the following:
	Discomfort
	Discomfort was highest for income collection as 65.2% of participants reported being somewhat or very uncomfortable. Discomfort was lowest for language information (6.6%). Minorities were less comfortable with the collection of preferred language but more comfortable disclosing income than non‐minorities. Females were less comfortable providing income than males. Participants of lower SES position were less comfortable with the collection of preferred language than those of higher position, but more comfortable reporting income. Participants over the age of 35 y reported more discomfort compared to the younger counterparts to the collection of ethnicity, language and income. Participants were least comfortable disclosing information through existing government records and most comfortable face to face with a physician.
	Quality of care
	60% of participants were at least somewhat concerned that the collection of this information could negatively affect their care. Minorities and females were more likely to hold concerns that the collection of sociodemographic information could negatively affect care.
Pinto et al. (2016)^19^	Eighteen of 50 who left a comment said that the survey was positive.
	Discomfort
	Some respondents (5 of 50 who left a comment, total of 407 respondents) reported feelings of discomfort in responding to the survey, especially to income. They felt that some of the questions were too personal or that they wanted to know that everyone would get the same standard of care no matter what.
Quan et al. (2006) 20	Overall, 84.8% felt comfortable recording their ethnicity in hospital charts. Reported harms include the following:
	Discomfort
	15% of participants reported discomfort providing their ethnicity in hospitals, with immigrants being the most uncomfortable.
Varcoe et al. (2009)^21^	Policy decision makers/leaders and health‐care workers viewed more positives than community leaders and patients. Reported harms include the following:
	Altered behaviour
	Many indicated that they would not answer questions related to ethnicity in a health‐care setting and would lie if they perceived their response would affect their treatment. Patient participants who identified themselves as aboriginal reported that they would alter their physical appearance such as dress.
	Discrimination
	Focus groups and patient participants anticipated the harm of being judged on the basis of assumptions and stereotypes. Many were concerned that the ethnicity data could influence health‐care staff to reinforce stereotypes that linked health behaviours to certain groups. Adding questions about ethnicity was viewed as a process that could fuel anxieties about inequities and that inequities could manifest in health care because of the negative perceptions or assumptions staff may have towards particular groups. Patients, focus group participants and some health‐care leaders identified concerns based on harmful discrimination, such as being treated rudely after identifying as aboriginal, that they had experienced or witnessed based on perceived socio‐economic status or ethnicity.
	Misuse and privacy concerns
	Participants also feared and questioned how the information collected might be used and for what aims.
	Offence/Negative reactions
	Felt offended if asked ethnicity. Patients who identified themselves as visible minorities felt anxiety, fear and anger. Concerns were related to further discrimination, marginalization and poorer care.
	Quality of care
	Focus group and patient participants believed that there was a possibility of receiving poorer care based on judgements from providing race/ethnicity information.
Wilson et al. (2013)^22^	Offence/Negative reactions
	Patients questioned why they were being asked race, ethnicity and language information. Some did not provide the information or were offended by the questions. Comments from patients included “I'm human” and “Can't you tell by looking at me?”

#### 4.3.1 Altered behaviour

In one study, patients indicated that they would alter their physical appearance, such as the way they dress, to hide their identities or to prevent being negatively judged in relation to ethnic stereotypes.^21^, ^30^ Patients reported that they lie about their race/ethnicity if they believe their response will affect their treatment.^21^

Another study found that the collection of sociodemographic information may affect care‐seeking behaviour, as 26.3% of Hispanics and 18.5% of African Americans in the study reported that they would be less likely go to a hospital or clinic collecting race/ethnicity information.^12^

#### 4.3.2 Discomfort

Discomfort was the most frequently reported harm perceived or experienced by patients.^12^, ^13^, ^14^, ^15^, ^16^, ^17^, ^18^, ^19^, ^20^, ^27^

Patients felt the most uncomfortable disclosing their income.^16^, ^18^, ^19^ Patients in two studies reported that the reason for discomfort was the belief that socio‐economic position should not determine immediate health‐care delivery.^16^, ^19^ One study found that non‐minorities, females, patients of high socio‐economic position and patients over the age of 35 years felt the most uncomfortable disclosing their income status.^18^

In three studies, 15% of patients indicated that they felt uncomfortable disclosing their race/ethnicity in a health‐care setting.^12^, ^13^, ^20^ Ethnic minorities and immigrants reported feeling the least comfortable providing their race/ethnicity information. For example, Baker et al.^13^ and Kandula et al^15^ found that Hispanics and Asians were more uncomfortable than whites, and Baker et al^12^ found that blacks were more uncomfortable than whites. Comfort was lower for patients who perceived discrimination in general or in medical care.^12^, ^15^

Patients reported being the least uncomfortable with the collection of language information.^16^, ^18^ However, participants from ethnic or cultural minorities were more likely to be uncomfortable disclosing their preferred language than non‐minorities.^16^, ^18^ Baker et al^13^ found that Hispanics (35.9%) were more likely to be uncomfortable than Chinese‐speaking Asians (13.8%). Other studies found that participants of lower socio‐economic position were less comfortable disclosing preferred language than those of higher position^18^ and males were less comfortable than females.^16^

Lofters et al^18^ found that patients over the age of 35 years reported more discomfort disclosing race/ethnicity/language compared to their younger counterparts, while Kirst et al^16^ found that patients 55 years of age and older were more uncomfortable compared to those aged 18‐34.

#### 4.3.3 Discrimination

Six studies reported that potential discrimination was a concern for patients.^12^, ^13^, ^14^, ^15^, ^16^, ^30^

Patients feared that the collection of income information could be used to judge, pity or discriminate against them.^16^ In one study, patients identified concerns based on harmful discrimination that they had experienced or witnessed based on socio‐economic status.^21^

Regarding the collection of religion information, Muslim patients reported feeling negatively stereotyped because of heightened awareness on terrorism.^14^

Patients reported being concerned that providing race/ethnicity or language information would be used to discriminate against them or other patients.^12^, ^13^ Other reasons were related to concerns that they would be judged negatively based on assumptions or stereotypes related to their race/ethnicity.^21^, ^30^ Non‐white and ethnic minority patients, including blacks and Hispanics, were most concerned about discrimination.^12^, ^13^ Kandula et al^15^ found that those who perceived discrimination in general and in medical care were more likely worried that their race/ethnicity information would be used to discriminate against them compared to those who did not perceive discrimination. These participants also worried that the government would use race/ethnicity information to find undocumented immigrants. Younger patients in this study were more likely to express concern for discrimination.^15^

#### 4.3.4 Misuse/Privacy concerns

There was a general concern by patients with regard to how the data would be used.^13^, ^15^, ^16^, ^21^ In one study, several patients were concerned with the confidentiality of collecting personal information and the security measures taken to protect privacy.^16^ Baker et al^13^ found that 38.5% of patients were very worried and 18.5% were somewhat worried that the government would use race/ethnicity/language information to find undocumented immigrants. The level of worry for the misuse of data was highest for Hispanics in two studies^13^, ^15^ and among younger patients (18‐34 years of age) and females in another.^16^

#### 4.3.5 Offence and Other negative reactions

Four studies reported that patients were offended when asked questions about race/ethnicity^21^, ^22^, ^27^ or language.^22^ In one study, when asked to report their race/ethnicity and language, patients replied “I'm human” and “can't you tell by looking at me?”.^22^

In another study, patients reported negative experiences providing race/ethnicity data, especially if they did not identify with any of the categories listed and were forced to choose “other.”^14^ In Jorgensen et al's^28^ study, patients reported being very upset that Hispanic or Latinos were not options and patients were required to choose either white or black. Patients were also dissatisfied about having to report ethnicity on repeat visits or without explanation about why these data were being collected.^14^, ^16^ One study reported that patients expressed anger, fear and anxiety with regard to being asked about their ethnicity, especially from participants who identified as members of visible minority groups.^21^, ^28^

#### 4.3.6 Quality of care

Three studies reported that patients felt that disclosing their race/ethnicity or income information may lead to poorer care as a result of judgements from health‐care providers.^16^, ^21^, ^28^, ^30^ One study found that 60% of patients were at least somewhat concerned that the collection of race/ethnicity or income information could affect their care with minorities and females being the most concerned.^18^

### 4.4 Providers’ perceptions of potential harms for patients

All seven studies reporting providers’ perceptions assessed the potential patient harms associated with the routine collection of race/ethnicity/culture/language data.^23^, ^24^, ^25^, ^26^, ^27^, ^28^, ^29^ None of the included studies assessed the providers’ perceptions of the potential harms associated with the collection of religion or income data. The potential harms described by providers are grouped using the following four themes: discomforting patients, discriminating against certain patients, misuse and privacy concerns, offending patients and provoking negative reactions, and quality of patient's care.

An overview of providers’ perceptions of the various harms for patients in each citation is presented in Table 4.

**Table 4:** Providers’ perceptions of potential harms for patients

Reference	Providers’ perceptions on outcomes for patients
Hasnain‐Wynia et al. (2004)^27^	70% of participating survey hospitals did not see any drawbacks. Reported harms perceived for their patients included the following:
	Offending patients/Negative reactions
	Participants reported a sense that patients might be insulted, offended or resist answering questions about their race and ethnicity.
	Quality of care
	There was a concern that patients would perceive their care to be different based on their race or ethnicity information. Participants were concerned that knowledge of patient's race and ethnicity would lead to segmenting service delivery, poorer care and discrimination. Participants felt patients would feel questions would signify that they will be treated differently than other patients. Participants felt patients would feel they would receive poorer care if they answer language and culture questions.
	Misuse and privacy concerns
	There was a fear that the race/ethnicity information collected would not remain confidential.
	Discriminating patients
	Participants noted the possibility that collecting data on race and ethnicity may be used to profile patients and discriminate them in the provision of care.
Hasnain‐Wynia et al. (2010)^23^	Misuse and privacy concerns
	Some practices believed that collection of race/ethnicity information could be a violation of privacy.
	Discomforting patients
	Some practices worried that asking questions about race/ethnicity or language could make patients uncomfortable.
Iqbal et al. (2012)^24^	69% of health‐care participants believed the collection of ethnicity data was important at a personal level, and 59% thought it was important at an organizational level. Reported harms perceived for their patients include the following:
	Offending patients/Negative reactions
	Staff feared being challenged by patients who wanted to know the reasons for the collection of ethnicity data and the possibility of ensuing hostility or offending patients.
Jorgensen et al. (2010)^28^	Hospital executives mentioned staff concerns more frequently than actual patient concerns (17 of 28 hospitals vs 9 of 28 hospitals, respectively). Reported harms perceived for their patients include the following:
	Offending patients/Negative reactions
	Staff concerns about potentially upsetting patients were frequently cited.
	Quality of patient's care
	Participants thought that patients would have questions on whether reporting race/ethnicity and language would impact their care.
	Discomforting patients
	Participants felt that patients might feel uncomfortable to answer questions about their race/ethnicity and language.
King et al. (2008)^25^	Offending patients/Negative reactions
	Participants expressed concern that patients will feel offended if asked race/ethnicity information in health‐care settings.
Nerenz et al. (2004)^26^	72% of participants that collected race/ethnicity data did not see any drawbacks to collecting the data. 44% of hospitals that did not collect race/ethnicity data did not see any drawbacks. Reported harms perceived for their patients include the following:
	Offending patients/Negative reactions
	Participants sensed that patients might be insulted or offended or resist answering questions about their race and ethnicity.
	Quality of patient's care
	There was a concern that patients will perceive their care will be different based on the race or ethnicity information. Participants felt patients would feel they were being treated differently from other patients. Concern that knowledge of race/ethnicity would lead to segmenting service delivery, discrimination and multiple standards of care.
	Misuse and privacy concerns
	There was a fear that the information collected would not remain confidential.
	Discriminating patients
	Participants also mentioned the possibility that collecting data on race and ethnicity might be used to profile patients and discriminate in the provision of care.
Thorlby et al. (2011)^29^	Offending patients/Negative reactions
	Staff held discomfort with asking patients about their race and ethnicity because they were concerned about negative reactions from patients.

#### 4.4.1 Discomforting patients

In two studies, health‐care providers reported concerns that asking questions about race/ethnicity/language could make patients uncomfortable or upset.^23^, ^28^

#### 4.4.2 Discrimination of patients

In two studies, there was a concern among health‐care providers that collecting data on race/ethnicity may be used to profile patients and discriminate against them in the provision of care.^26^, ^27^ In one study, health‐care providers identified concerns based on harmful discrimination that they had experienced or witnessed based on socio‐economic status.^21^

#### 4.4.3 Misuse and privacy concerns

In three studies, health‐care providers reported being concerned that patient's race/ethnicity information would not remain confidential or that collecting this information could violate a patient's privacy.^23^, ^26^, ^27^

#### 4.4.4 Offending patients and provoking negative reactions

In four studies, health‐care providers reported that patients might be insulted, offended or resist answering questions about their race/ethnicity.^24^, ^25^, ^26^, ^27^ Health‐care providers in two studies reported concerns about being challenged by patients who want to know the reasons for the collection of race/ethnicity data and worried that this may lead to hostility or negative reactions in patients.^24^, ^29^

#### 4.4.5 Quality of patient's care

In three studies, health‐care providers reported being concerned that patients would perceive their care to be different or be worry that they would be treated differently based on their reported race/ethnicity/culture/language.^26^, ^27^, ^29^ Two studies also reported that health‐care providers feared that this information would lead to segmenting service delivery and poorer care for their patients.^26^, ^27^

### 4.5 Best Practices: Patient recommendations for sociodemographic data collection

Five studies included patients’ recommendations for the collection of sociodemographic data.^12^, ^13^, ^14^, ^16^, ^18^ An overview of patients’ recommendations for collecting sociodemographic data in each citation is presented in Table 5.

**Table 5:** Data summary table: Patients’ recommendations for collecting sociodemographic data

Reference	Best practices: Reducing patient harms
Baker et al. (2005)^12^	Who should collect/see data
	For participants who reported that they were not fully comfortable providing race/ethnicity information to a clerk, they reported feeling more comfortable providing this information to a doctor (54.4%) or nurse (42.0%).
	Need for collection
	96.8% of participants somewhat or strongly agreed that hospitals and clinics should conduct studies to ensure that all patients get the same quality of care regardless of race/ethnicity.
	Statement increasing comfort
	Comfort levels increased when participants heard the statement: “We want to make sure that all our patients get the best care possible, regardless of their race or ethnic background. We would like you to tell us your race or ethnic background so that we can review the treatment that all patients receive and make sure that everyone gets the highest quality of care.”
	Statement decreasing comfort
	Mean comfort levels decreased for non‐white participants after hearing the statements: “Several government agencies recommend that we collect information on the race and ethnic backgrounds of our patients as part of a national effort to make sure all patients have access to quality health care. Please tell me your race or ethnic background,” and “We take care of patients from many different backgrounds. We would like you to tell us your race or ethnic background so that we can understand our patients better. This will help us decide who to hire, how to train our staff better, and what health information is most helpful for our patients.”
Baker et al. (2007)^13^	Who should collect/see data
	42.3% of participants somewhat or strongly agreed that doctors, nurses and other health‐care workers should not see the collected race/ethnicity information. 22% of participants were unsure. Blacks, Latinos and Chinese were more likely than whites to agree that providers should not see these data.
	Need for collection
	87.8% of participants somewhat or strongly agreed that hospitals and clinics should conduct studies to ensure that all patients get the same quality of care regardless of race/ethnicity.
	Statement increasing comfort
	Comfort levels increased the most when participants heard the statement: “We take care of patients from many different backgrounds. Please tell me your race or ethnic background so we can understand more about the patients we serve. This will help us train our staff better and improve our health education materials.” The magnitude of comfort was higher for Spanish‐speaking Latinos and Chinese‐speaking Asians.
	Statement decreasing comfort
	Comfort decreased for approximately one‐third of participants after hearing the statement: “We want to make sure all our patients get the best care possible. We would like you to tell us your race or ethnic background so we can review the treatment that patients receive and make sure everyone gets the highest quality care. Only a few people here will be able to see this information. The doctors and nurses caring for you will not be given this information.”
Iqbal et al. (2012)^14^	Who should collect/see data
	Participants believed that general practitioners should be collecting the information.
	When to collect data
	There was a strong belief that the information should not be collected every visit as ethnicity is unlikely to change.
	Need for collection
	They indicated that health‐care settings should clearly explain the need for collection, the benefits, how data will be used and how it will be kept secret/confidential.
Kirst et al. (2013)^16^	Who should collect/see data
	29% of survey participants indicated that they were most comfortable providing sociodemographic information face to face with a family physician, 22% face to face with a hospital clerk and 20% on a form in a hospital, 14% survey through mail or Internet and 12% disclosure through existing government records. 3% indicated none of the above. Interview participants also indicated that their preferred method to disclose sociodemographic information would be face to face with a family physician due to the ongoing relationship and trust.
	When to collect data
	Interview participants indicated that they would prefer that personal characteristics would not be asked at every health‐care visit.
	Need for collection
	Interview participants were more open to disclosing information if there was sufficient explanation for the use of the information. Simply saying the information is needed is not enough patients should see how the information is being used, how it benefits them personally and the population as a whole, security and privacy measures taken related to the use and storage and confidentiality. They believed that an educational campaign would be beneficial explaining the purpose of the information collection, use, security and privacy.
Lofters et al. (2011)^18^	Who should collect/see data
	Comfort level for the collection of information was the highest for face‐to‐face interviews with a family physician (67.7%), followed by form in a hospital (49.3%), face to face with a hospital clerk (47.6%), survey by mail or on the Internet (31.3%) and accessing information from existing government records (28.6%). 5.6% indicated none of the above.

#### 4.5.1 Who should collect and see sociodemographic data

Patients in four studies reported that they would feel most comfortable disclosing their sociodemographic information face to face to a doctor, preferably to a family physician.^12^, ^14^, ^16^, ^18^ However, one study found that 42.3% of patients somewhat or strongly agreed that doctors, nurses or other health‐care workers should not see the race/ethnicity information and 22% were unsure.^13^ Blacks, Latinos and Chinese were more likely than whites to agree that providers should not see these data.^13^

#### 4.5.2 When to collect sociodemographic data

There was a strong belief among patients in two studies that sociodemographic information should not be collected at every visit^14^, ^16^ since some of these characteristics (eg ethnicity) are unlikely to change.^14^

#### 4.5.3 Describing the need for sociodemographic data collection

Baker et al and Baker et al found that nearly all patients agreed that hospitals and clinics should conduct studies to ensure that all patients get the same quality of care regardless of race/ethnicity (96.8% and 87.8%, respectively).^12^, ^13^ Patients stated that it was important for health‐care settings to clearly explain the need for collecting sociodemographic information, the benefits of collecting these data, how the data will be used and how the data will be kept secure and confidential.^14^, ^16^ Participants in one study reported that an educational campaign would be helpful to explain these factors.^16^

Two studies assessed changes in patient's comfort levels after hearing an explanation of the reasons for sociodemographic collection.^12^, ^13^ One study conducted by Baker et al found that mean comfort levels increased when reasons for data collection were provided with the following statement: “We want to make sure that all our patients get the best care possible, regardless of their race or ethnic background. We would like you to tell us your race or ethnic background so that we can review the treatment that all patients receive and make sure that everyone gets the highest quality of care.” Comfort levels decreased for non‐white participants when the reasons for race/ethnicity data collection were described with this statement: “We take care of patients from many different backgrounds. We would like you to tell us your race or ethnic background so that we can understand our patients better. This will help us decide who to hire, how to train our staff better, and what health information is most helpful for our patients.” However, a later study conducted by Baker et al^13^ found that comfort levels increased when race/ethnicity data collection was related to needs assessment using the statement: “We take care of patients from many different backgrounds. Please tell me your race or ethnic background so we can understand more about the patients we serve. This will help us train our staff better and improve our health education materials.” The magnitude of change was highest for Spanish‐speaking Latinos and Chinese‐speaking Asians. The authors postulated that patients in the first study may have reacted negatively to the original needs assessment statement since it may have indicated that the data were being collected to meet hiring quotas.^13^

## 5 DISCUSSION

The findings from this review demonstrate that although the majority of patients support the routine collection of sociodemographic data in a health‐care setting, patient harms are possible. Fifteen of the studies reported on perceptions, while seven reported on actual experienced harms reported by patients. Commonly cited harms perceived or experienced by patients included altered behaviour which may affect care‐seeking, a belief that care will be different, concerns about the misuse or privacy of the information, discomfort, fear of discrimination, and offence or other negative reactions.

Comfort levels for the collection of sociodemographic data varied among types of sociodemographic data collected. Patients were the most comfortable providing language information and least comfortable providing income information. Expanding the response ranges may help make patients feel more comfortable reporting their income.

Perceived and experienced harms differed across population subgroups. Minority patients perceived or experienced more harms when disclosing sociodemographic information and had lower trust in their health‐care provider than whites, making them vulnerable to health inequities.^12^ The studies reported that these harms likely stem from the experienced discrimination faced by racial/ethnic minorities.

Fixed categories related to race/ethnicity assume that patients can fit themselves into one particular category and may isolate those who do not identify with any of the categories offered.^31^ Using an open‐ended question for the collection of race/ethnicity information may help to alleviate these concerns and reduce the rates of missing or unusable data.^19^, ^31^

The studies included in this review suggest that harms may be mitigated by sufficiently explaining the need for and benefits of collecting sociodemographic data, how the data will be used and how the data will be kept secure and confidential. An educational campaign may help to address these factors. Additionally, participants in the included studies reported that they would prefer to disclose their sociodemographic information face to face to a doctor.

Health‐care providers reported similar concerns with the collection of sociodemographic information as patients. The studies that assessed health‐care providers’ opinions on potential harms focused on race/ethnicity/culture/language; it is likely that the same concerns apply for the collection of religion and income data.^32^ Health‐care providers may not be fully prepared to ask their patients about sociodemographic information and to address patient concerns with this data collection. It may be helpful for health‐care organizations provide their staff with the skills and tools needed to appropriately collect sociodemographic information.^31^

Our rapid review has some important limitations. The studies included in our review had a higher proportion of white and English‐speaking participants. It is possible that their attitudes are different than other population groups. In addition, we searched a limited number of databases to identify relevant studies. It is possible that the search did not capture all relevant studies. However, we used broad inclusion criteria and searched both the references of included studies and their related citations. Although we did not restrict our search to English language studies, we only included English papers which may limit the generalizability of our findings to other non‐English settings. Finally, we did not assess the risk of bias of the studies included in this rapid review. The study designs included in this review were almost all descriptive studies utilizing survey methods and are therefore likely to have a high risk of bias. Overall, most of the included studies had a large sample size, but the reported non‐response rate of studies was relatively high, ranging from 27.3% to 96.9%.^18^, ^29^ However, it is possible that patients who were opposed to participating were more likely to be opposed to the collection of sociodemographic information due to negative attitudes or experiences. This would likely cause our results to underestimate the potential harms. Additionally, the majority of the studies included in this review reported only on perceived harms (12 studies), while three studies reported both perceived and experienced harms and only four studies focused on harms experienced by patients or providers.

The results of this review provide insight into the potential harms perceived or experienced by patients and the concerns of health‐care providers with regard to the routine collection of race/ethnicity/culture/language, religion and income data. We have also identified some recommended practices for how, when and by whom these data should be collected. The results of this review can be used to inform the design of data collection procedures, including who asks for the information, how the information is categorized and under what circumstances it is collected. Further research is needed to explore how perceived potential harms relate to actual harms experienced by patients as well as strategies to reduce the risk of patient discomfort and distress with providing this information.

## 6 CONCLUSION

The collection of sociodemographic data, notably race/ethnicity/culture/language, religion and income, is necessary to guide clinical decisions and reduce health inequities. Although the studies included in this review suggest that the public generally supports the collection of sociodemographic information, there are potential harms associated with collecting this information in a health‐care setting. The associated harms, both perceived and experienced, were most pronounced for minority population subgroups and for the collection of income information. More research is needed on strategies to overcome the potential harms associated with collecting race/ethnicity/culture/language, religion and income data in a health‐care setting. However, the studies included in this review indicate that harms may be mitigated by sufficiently educating health‐care providers and patients on the reasons for the collection of this information.

## CONFLICTS OF INTEREST

None to declare.

## References

[1] Marmot M , Friel S , Bell R , Houweling TAJ , Taylor S , Hlt CSD . Closing the gap in a generation: health equity through action on the social determinants of health. Lancet. 2008;372(9650):1661‐1669. 18994664 10.1016/S0140-6736(08)61690-6

[2] Whitehead M . The concepts and principles of equity and health. Int J Health Serv. 1992;22(3):429‐445. 1644507 10.2190/986L-LHQ6-2VTE-YRRN

[3] Evans T , Brown H . Road traffic crashes: operationalizing equity in the context of health sector reform. Inj Control Saf Promot. 2003;10(1–2):11‐12. 12772480 10.1076/icsp.10.1.11.14117

[4] Oliver S , Kavanagh J , Caird J , Lorenc T , Oliver K , Harden A . Health promotion, inequalities and young people's health. A systematic review of research; 2008 https://eppi.ioe.ac.uk/cms/Default.aspx?tabid=2410. Accessed September 7, 2018.

[5] O'Neill J , Tabish H , Welch V , et al. Applying an equity lens to interventions: using PROGRESS ensures consideration of socially stratifying factors to illuminate inequities in health. J Clin Epidemiol. 2014;67(1):56‐64. 24189091 10.1016/j.jclinepi.2013.08.005

[6] LaForge K , Gold R , Cottrell E , et al. How 6 organizations developed tools and processes for social determinants of health screening in primary care: an overview. J Ambul Care Manage. 2018;41(1):2‐14. 28990990 10.1097/JAC.0000000000000221 PMC5705433

[7] Wray R , Agic B , Bennett‐AbuAyyash C , et al. We ask because we care: The Tri‐Hospital + TPH Health Equity Data Collection Research Project Report. Toronto, ON: Tri‐Hospital + TPH Steering Committee; 2013.

[8] Iqbal G , Gumber A , Johnson M , Szczepura A , Wilson S , Dunn JA . Improving ethnicity data collection for health statistics in the UK. Divers Heal Care. 2009;6(4):267‐285.

[9] Garritty CM , Norris SL , Moher D . Developing WHO rapid advice guidelines in the setting of a public health emergency. J Clin Epidemiol. 2017;82:47‐60. 27591906 10.1016/j.jclinepi.2016.08.010 PMC7125868

[10] Khangura S , Konnyu K , Cushman R , Grimshaw J , Moher D . Evidence summaries: the evolution of a rapid review approach. Syst Rev. 2012;1:10. 22587960 10.1186/2046-4053-1-10 PMC3351736

[11] Higgins JPT, Green S (eds). Cochrane Handbook for Systematic Reviews of Interventions. London: The Cochrane Collaboration; 2011.

[12] Baker DW , Cameron KA , Feinglass J , et al. Patients’ attitudes toward health care providers collecting information about their race and ethnicity. J Gen Intern Med. 2005;20(10):895‐900. 16191134 10.1111/j.1525-1497.2005.0195.x PMC1490236

[13] Baker DW , Hasnain‐Wynia R , Kandula NR , Thompson JA , Brown ER . Attitudes toward health care providers, collection information about patient's race, ethnicity, and language. Med Care. 2007;2007(45):11. 10.1097/MLR.0b013e318127148f 18049343

[14] Iqbal G , Johnson MR , Szczepura A , Wilson S , Gumber A , Dunn JA . UK ethnicity data collection for healthcare statistics: the South Asian perspective. BMC Public Health. 2012;12:243. 22452827 10.1186/1471-2458-12-243 PMC3339513

[15] Kandula NR , Hasnain‐Wynia R , Thompson JA , Brown ER , Baker DW . Association between prior experiences of discrimination and patients’ attitudes towards health care providers collecting information about race and ethnicity. J Gen Intern Med. 2009;24(7):789‐794. 19415392 10.1007/s11606-009-0991-z PMC2695532

[16] Kirst M , Shankardass K , Bomze S , Lofters A , Quinonez C . Sociodemographic data collection for health equity measurement: a mixed methods study examining public opinions. Int J Equity Health. 2013;12:75. 24119260 10.1186/1475-9276-12-75 PMC3766029

[17] Lee WC , Veeranki SP , Serag H , Eschbach K , Smith KD . Improving the collection of race, ethnicity, and language data to reduce healthcare disparities: a case study from an academic medical center. Perspect Health Inf Manag. 2016;13(Fall):1 g. PMC5075235 27843424

[18] Lofters AK , Shankardass K , Kirst M , Quinonez C . Sociodemographic data collection in healthcare settings: an examination of public opinions. Med Care. 2011;49(2):193‐199. 21150797 10.1097/MLR.0b013e3181f81edb

[19] Pinto AD , Glattstein‐Young G , Mohamed A , Bloch G , Leung FH , Glazier RH . Building a foundation to reduce health inequities: routine collection of sociodemographic data in primary care. J Am Board Fam Med. 2016;29(3):348‐355. 27170792 10.3122/jabfm.2016.03.150280

[20] Quan H , Wong A , Johnson D , Ghali WA . The public endorses collection of ethnicity information in hospital: implications for routine data capture in Canadian health systems. Healthc Policy. 2006;1(3):55‐64. PMC2585343 19305671

[21] Varcoe C , Browne AJ , Wong S , Smye VL . Harms and benefits: collecting ethnicity data in a clinical context. Soc Sci Med. 2009;68(9):1659‐1666. 19286294 10.1016/j.socscimed.2009.02.034

[22] Wilson G , Hasnain‐Wynia R , Hauser D , Calman N . Recommendations on collection of patient race, ethnicity, and language data in a community health center. J Health Care Poor Underserved. 2013;2013(24):2. 10.1353/hpu.2013.0071 23728053

[23] Hasnain‐Wynia R , Van Dyke K , Youdelman M , et al. Barriers to collecting patient race, ethnicity, and primary language data in physician practices: an exploratory study. J Natl Med Assoc. 2010;102(9):769‐775. 20922920 10.1016/s0027-9684(15)30673-8

[24] Iqbal G , Johnson MRD , Szczepura A , Gumber A , Wilson S , Dunn JA . Ethnicity data collection in the UK: The healthcare professional's perspective. Dicers Equal Heal Care. 2012;9(4):281‐290.

[25] King RK , Green AR , Tan‐McGrory A , Donahue EJ , Kimbrough‐Sugick J , Betancourt JR . A plan for action: key perspectives from the racial/ethnic disparities strategy forum. Milbank Q. 2008;86(2):241‐272. 18522613 10.1111/j.1468-0009.2008.00521.x PMC2690363

[26] Nerenz D , Currier C . Collection of data on race and ethnicity by private‐sector organizations: Hospitals, health plans, and medical groups In: ver Ploeg M, Perrin E, eds. Eliminating Health Disparities: Measurement and Data Needs. Washington, DC: The National Academies Press; 2004:249‐271. 25009872

[27] Hasnain‐Wynia R , Pierce D , Pittman MA , Trust HRaE . Who, when, and how: The current state of race, ethnicity, and primary language data collection in hospitals. The Commonwealth Fund; 2004.

[28] Jorgensen S , Thorlby R , Weinick RM , Ayanian JZ . Responses of Massachusetts hospitals to a state mandate to collect race, ethnicity and language data from patients: a qualitative study. BMC Health Serv Res. 2010;10:352. 21194450 10.1186/1472-6963-10-352 PMC3022878

[29] Thorlby R , Jorgensen S , Siegel B , Ayanian JZ . How health care organizations are using data on patients’ race and ethnicity to improve quality of care. Milbank Q. 2011;89(2):226‐255. 21676022 10.1111/j.1468-0009.2011.00627.x PMC3142338

[30] Browne AJ , Varcoe CM , Wong ST , Smye VL , Khan KB . Can ethnicity data collected at an organizational level be useful in addressing health and healthcare inequities? Ethn Health. 2014;19(2):240‐254. 23909292 10.1080/13557858.2013.814766

[31] Hasnain‐Wynia R , Baker DW . Obtaining data on patient race, ethnicity, and primary language in health care organizations: current challenges and proposed solutions. Health Serv Res. 2006;41(4 Pt 1):1501‐1518. 16899021 10.1111/j.1475-6773.2006.00552.x PMC1797091

[32] Tarrant C , Wobi F , Angell E . Tackling health inequalities: socio‐demographic data could play a bigger role. Fam Pract. 2013;30(6):613‐614. 24249332 10.1093/fampra/cmt071

