# Supplementary material for: Potential harms associated with routine collection of patient sociodemographic information: A rapid review
Source: Health Expect. 2018 Oct 19;22(1):114–29. doi: 10.1111/hex.12837 (PMC6351414; doi:10.1111/hex.12837)
Supplement: Supplementary file 1 [file HEX-22-114-s001.docx]

**Appendix 1: MEDLINE Search Strategy**

Database: Ovid MEDLINE(R) Epub Ahead of Print, In-Process & Other Non-Indexed Citations, Ovid

MEDLINE(R) Daily and Ovid MEDLINE(R) <1946 to Present>

Search Strategy:

--------------------------------------------------------------------------------

1 "Delivery of Health Care"/es, mt, st, sn [Ethics, Methods, Organization & Administration,

Standards, Statistics & Numerical Data] (16127)

2 Data Collection/es, mt, og, st, sn [Ethics, Methods, Organization & Administration,

Standards, Statistics & Numerical Data] (15214)

3 Interviews as Topic/mt, st [Methods, Standards] (3293)

4 "Surveys and Questionnaires"/mt, st, ut [Methods, Standards, Utilization] (11989)

5 "Outcome and Process Assessment (Health Care)"/es, mt, og, st, sn [Ethics, Methods,

Organization & Administration, Standards, Statistics & Numerical Data] (5247)

6 Health Services/es, mt, st, sn [Ethics, Methods, Standards, Statistics & Numerical Data]

(2454)

7 *Primary Health Care/es, mt, og, st, sn, td [Ethics, Methods, Organization & Administration,

Standards, Statistics & Numerical Data, Trends] (20987)

8 ((obtain$ or gather$ or input$ or report$ or self report$ or record$ or identif$ or collect$)

and (data or information or detail$)).ti. (20429)

9 or/1-8 (91794)

10 *Socioeconomic Factors/ (10666)

11 *Social Determinants of Health/ (838)

12 *Ethnic Groups/ (27232)

13 demographic.ti,ab. (186737)

14 sociodemographic.ti,ab. (34345)

15 (ethnic$ or race or racial or religio$ or cultur$ or language$ or minorit$ or refugee or

indigenous or aboriginal or African american).ti. (320185)

16 (income or socioeconomic$ or socio-economic$ or social).ti,ab. (560450)

17 Continental Population Groups/ (19098)

18 *INCOME/ (7995)

19 *RELIGION/ (5934)

20 or/10-19 (1057396)

21 "Attitude of Health Personnel"/ (108574)

22 Health Status Disparities/ or Health Services Accessibility/ (73760)

23 Healthcare Disparities/ (12226)

24 Health Equity/ (349)

25 Prejudice/ or Social Discrimination/ (24409)

26 (adverse adj (events or effects)).tw. (213219)

27 (inequit$ or equit$ or inequal$ or equal$).tw. (369625)

28 disadvantage$.tw. (64616)

29 (social adj2 problem$).tw. (9767)

30 harm$.tw. (144595)

31 poverty.tw. (20373)

32 ethic$.ti,ab. (104299)

33 or/21-32 (1074006)

34 9 and 20 and 33 (3033)
